# Potential effects of commonly applied drugs on neural stem cell proliferation and viability: A hypothesis-generating systematic review and meta-analysis

**DOI:** 10.3389/fnmol.2022.975697

**Published:** 2022-10-05

**Authors:** Katherine R. H. Mortimer, Hannah Vernon-Browne, Marietta Zille, Nadine Didwischus, Johannes Boltze

**Affiliations:** ^1^School of Life Sciences, University of Warwick, Coventry, United Kingdom; ^2^Faculty of Biological Sciences, University of Leeds, Leeds, United Kingdom; ^3^Division of Pharmacology and Toxicology, Department of Pharmaceutical Sciences, University of Vienna, Vienna, Austria; ^4^Department of Radiology, University of Pittsburgh, Pittsburgh, PA, United States; ^5^Center for the Neural Basis of Cognition and Center for Neuroscience, McGowan Institute for Regenerative Medicine, Pittsburgh, PA, United States

**Keywords:** neural stem cells, antihypertensives, statins, proliferation, differentiation, viability, drug effects, drug cell interaction

## Abstract

Neural stem cell (NSC) transplantation is an emerging and promising approach to combat neurodegenerative diseases. While NSCs can differentiate into neural cell types, many therapeutic effects are mediated by paracrine, “drug-like” mechanisms. Neurodegenerative diseases are predominantly a burden of the elderly who commonly suffer from comorbidities and thus are subject to pharmacotherapies. There is substantial knowledge about drug-drug interactions but almost nothing is known about a potential impact of pharmacotherapy on NSCs. Such knowledge is decisive for designing tailored treatment programs for individual patients. Previous studies revealed preliminary evidence that the anti-depressants fluoxetine and imipramine may affect NSC viability and proliferation. Here, we derive a hypothesis on how commonly applied drugs, statins and antihypertensives, may affect NSC viability, proliferation, and differentiation. We conducted a systematic review and meta-analysis looking at potential effects of commonly prescribed antihypertensive and antihyperlipidemic medication on NSC function. PubMed and Web of Science databases were searched on according to the Preferred Reporting Items for Systematic reviews and Meta-Analyses (PRISMA) guidelines. Publications were assessed against *a priori* established selection criteria for relevancy. A meta-analysis was then performed on data extracted from publications eligible for full text review to estimate drug effects on NSC functions. Our systematic review identified 1,017 potential studies, 55 of which were eligible for full text review. Out of those, 21 were included in the qualitative synthesis. The meta-analysis was performed on 13 publications; the remainder were excluded as they met exclusion criteria or lacked sufficient data to perform a meta-analysis. The meta-analysis revealed that alpha-2 adrenoceptor agonists, an anti-hypertensive drug class [*p* < 0.05, 95% confidence intervals (CI) = –1.54; –0.35], and various statins [*p* < 0.05, 95% CI = –3.17; –0.0694] had an inhibiting effect on NSC proliferation. Moreover, we present preliminary evidence that L-type calcium channel blockers and statins, particularly lovastatin, may reduce NSC viability. Although the data available in the literature is limited, there are clear indications for an impact of commonly applied drugs, in particular statins, on NSC function. Considering the modes of action of the respective drugs, we reveal plausible mechanisms by which this impact may be mediated, creating a testable hypothesis, and providing insights into how future confirmative research on this topic may be conducted.

## Introduction

Neural stem cells (NSCs) are self-renewing, multipotent stem cells. They are found in distinct germinal niches in the mammalian brain, predominantly in the subventricular zone or the subgranular zone in the dentate gyrus of the hippocampus (Leal-Galicia et al., 2021; Moreno-Jiménez et al., 2021). Due to their ability to differentiate into neurons and glia cells, NSCs have been considered as a valuable resource to combat acute and subacute neurodegenerative diseases for which causal treatments are mostly unavailable. Interestingly, NSCs also exert at least some of their therapeutic benefits by paracrine mechanisms (Janowski et al., 2015).

However, the number of endogenous NSCs in the human brain is too small to provide benefits after substantial brain damage and there is evidence that NSCs are susceptible to DNA damage as well as damage by reactive oxygen species, both of which are associated with aging and age-related diseases (Limke and Rao, 2002). This means that NSCs used for transplantation should be ideally provided from exogenous sources and NSCs are indeed effective after both local and systemic transplantation in preclinical studies (Rosenblum et al., 2012; Mine et al., 2013; Yoon et al., 2020). First clinical trials have been initiated to assess the safety and therapeutic potential of NSC products in the clinical arena (Kalladka et al., 2016; Zhang et al., 2019; Muir et al., 2020).

Patients suffering from common neurodegenerative diseases are often of advanced age and exhibit comorbidities such as hypertension and hyperlipidemia, which are frequent among the elderly. Established pharmacotherapies are applied to control these comorbidities. Although substantial knowledge is available on drug-drug interactions, almost nothing is known about potential drug-NSC interactions. NSCs could become exposed to drugs after systemic administration, but also upon local, stereotactic administration as the blood-brain barrier is often locally compromised in neurodegenerative diseases.

It is known that some selective serotonin reuptake inhibitors, in particular fluoxetine, promote NSC proliferation. This which was confirmed in a recent meta-analysis (Ikhsan et al., 2019). Potential mechanisms explaining the effect have been suggested and the meta-analysis also revealed limited, preliminary indications for potential other drug-mediated effects on NSC characteristics. Indeed, it is reasonable to assume that at least some effects of drugs on NSCs exist, which could both promote or impair core NSC characteristics such as viability, proliferation, neuronal differentiation, and migration (Boltze et al., 2015). These effects would be particularly relevant for commonly applied drugs. Importantly, knowledge about such influences would be crucial for designing treatment strategies tailored to individual patients and their pharmacotherapy profiles or may be relevant for future clinical trial design.

### Statement of hypothesis

We hypothesized that drug classes frequently applied to treat common conditions in the elderly, in particular anti-hypertensives and anti-dyslipidemics, can affect at least one of the four NSC characteristics, i.e., proliferation, differentiation, viability, and migration. We further assumed that knowledge on such effects would be scattered in the literature and would mostly be presented as secondary findings.

### Collection of data to investigate the hypothesis

We conducted a systematic review and meta-analysis to collect relevant information from the literature to investigate our hypothesis. The systematic review was carried out according to the Preferred Reporting Items for Systematic reviews and Meta-Analysis (PRISMA) guidelines (Moher et al., 2009).

### Search strategy and selection criteria

Publications listed on PubMed and Web of Science were searched using a pre-defined systematic search strategy comprising three elements (see Supplementary Table 1). In brief, the first element comprised the most commonly prescribed antihypertensive medications and statins (National Health Service United Kingdom, 2018; National Institute for Health and Care Excellence guidelines, 2019), including both individual drug names as well as substance classes (e.g., beta blockers (BBs)). The second element of the search strategy then represented synonyms for NSCs, as well as terms describing “downstream” cell populations such as neural and neuronal precursors. The third element comprised terms describing the four main NSC characteristics, proliferation, neuronal differentiation, viability, and migration. Search terms in all elements were combined with OR whereas elements were combined with AND. The search was conducted by two investigators (KRHM, HV-B). In cases of doubt, senior investigators (ND and JB) were consulted for clarification.

### Exclusion and inclusion criteria

Reviews and publications written in languages other than English were excluded. We further excluded studies using cells other than NSCs or those that did not measure any direct drug effects on NSCs, studies using drugs exclusively as part of an experimental procedure (for instance in *in vivo* experiments), and those subjecting NSCs to additional stressors such as hypoxia. Publications for which full texts were not available were also excluded.

### Literature screening and data extraction

The search was conducted in 2021, and scientific articles published between January 1991 and December 2020 were included. All publications from both databases were gathered, and duplicates were removed. Then, all titles were screened for relevancy, followed by screening the abstracts. After elimination of publications by title and abstract screening, full texts were analyzed and excluded based on exclusion criteria. In case included publications reported multiple experiments (for instance using different drugs or drug concentrations), all these experiments were considered as individual records from that study. Thus, the number of experiments included in the analysis is higher than the number of publications retrieved by the systematic literature search.

### Risk of bias assessment

We assessed the potential risk of bias in the included studies according to the Cochrane guidelines (Higgins et al., 2022), with modifications for preclinical research (Office of Health Assessment and Translation, 2019), considering the following: (i) randomization, (ii) sample size calculation, (iii) allocation concealment (all selection bias), (iv) blinding, (v) exposure classification (i.e., target engagement, verification of compound) (both performance/detection bias), (vi) complete outcome analysis (attrition bias), (vii) selective reporting (reporting bias), (viii) conflict of interest, and (ix) correct statistical analysis (both other bias). The assessment was performed with respect to the effects of commonly applied drugs on neural stem cell proliferation, viability, differentiation, and migration. Each study was rated “low risk,” “high risk,” or “unclear risk,” according to the Cochrane guidelines. “Unclear risk” was judged either due to lack of information or uncertainty over the potential for bias (Higgins et al., 2022).

### Meta-analysis

We used standardized mean difference (SMD), where the mean difference in each reported experiment was divided by the standard deviation. This was to account for the heterogeneity between methods used to obtain the data in each publication. We chose to use the Hedge’s g SMD coefficient to calculate effect sizes due to the small sample sizes. The data was split into NSC characteristics and drug class. From the data extracted, we generated forest plots using the Camarades meta-analysis tool^1^ and obtained the *p* values and 95% confidence intervals (CI).

## Results

### Systematic search and meta-analysis results

**FIGURE 1:**
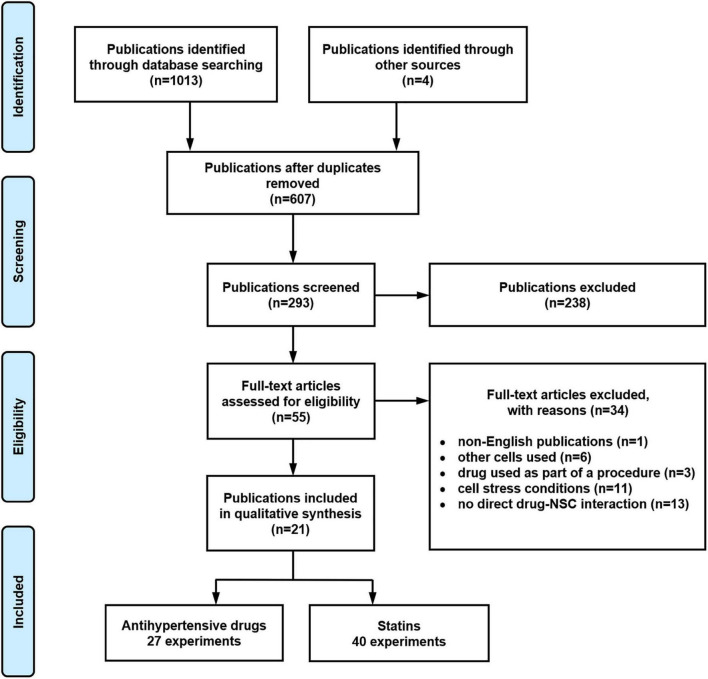
Systematic literature search flow diagram illustrating the results reported according to the Preferred Reporting Items for Systematic reviews and Meta-Analyses (PRISMA) guidelines.

Figure 1 provides details regarding the systematic search. After the removal of 410 duplicates, 607 publications were identified, and their titles screened for relevancy. This removed 314 publications, leaving 293 publications to be screened for abstract relevancy. This resulted in 238 publications being excluded, with 55 publications to undergo full text analysis. Application of the predefined exclusion criteria resulted in 34 excluded publications. From the remaining 21 publications, 67 experiments were extracted, with 27 experiments reporting the effect of anti-hypertensive drugs and 40 experiments reporting the effect of statins on a NSC function.

For the meta-analysis, 13 publications reporting 54 experiments were identified with sufficient data to conduct the meta-analysis including sample size, mean, and standard deviation/error. Eight experiments were excluded from the meta-analysis due to incomplete information provided. There was insufficient data for a meta-analysis of drug effects on NSC migration as there was only one experiment on statin effect and two on the effect of α2-adrenergic receptor agonists on NSC migration. There were also only two experiments on the effect of α2-adrenergic receptor agonists on NSC neuronal differentiation and, therefore, these experiments have also been excluded from the meta-analysis results.

### Number of experiments investigating drug effects on neural stem cell characteristics

Table 1 provides an overview of the experiments investigating the impact of respective antihypertensive drugs on NSC characteristics. The effects of calcium channel blockers (CCBs) and α2-adrenergic receptor agonists (α2As) on NSC characteristics were most frequently investigated (12 experiments and 10 experiments, respectively). The effects of BBs were assessed in four experiments. Angiotensin 2 receptors antagonists (A2Ras) were only investigated in one experiment, and no experiments assessed angiotensin-converting enzyme (ACE) inhibitor effects on NSCs.

**TABLE 1:** Overview of experiments investigating effects of antihypertensive drugs on neural stem cell (NSC) characteristics.

Drug class	Proliferation	Differentiation	Viability	Migration	Total
Calcium channel blockers (L-type)	1	4	7	0	12
Angiotensin 2 receptor antagonists	1	0	0	0	1
Alpha-2 adrenoceptor agonists	7	2	0	2	10
Beta blockers	3	1	0	0	4
Angiotensin converting enzyme inhibitors	0	0	0	0	0
Total	12	7	7	2	27

Statin effects on NSC characteristics were investigated more frequently than that of antihypertensive drugs with a total of 40 experiments being reported (Table 2). The effects of simvastatin and lovastatin were investigated in 14 and 13 experiments, respectively, followed by pravastatin (12 experiments). Only one experiment investigated the effects of atorvastatin on NSCs.

**TABLE 2:** Overview of experiments investigating statin effects on neural stem cell (NSC) characteristics.

Drug	Proliferation	Differentiation	Viability	Migration	Total
Lovastatin	0	0	13	0	13
Atorvastatin	1	0	0	0	1
Pravastatin	6	1	5	0	12
Simvastatin	5	3	5	1	14
Total	12	4	23	1	40

The most frequently investigated NSC characteristic was viability, with 30 experiments overall, 23 experiments reported the effects of statins and seven experiments those of CCBs on NSC viability. The second most frequently reported characteristic was NSC proliferation, with 24 experiments in total, 12 on statins, seven on α2As, three on BBs, and one each on CCBs and A2Ras. Migration was the least observed NSC characteristic with only one experiment on statins and two on α2As.

### Overview of drug effects on neural stem cell characteristics

Table 3 provides an overview of drug effects on NSC characteristics as well as the number of experiments from which such data has been reported. There was great heterogeneity within and between drug classes, and often no data about potential effects of a drug on a particular NSC characteristic was available in the literature. Nevertheless, we were able to obtain some interesting information from the available data. For instance, five experiments reported that α2As inhibit proliferation, apart from prazosin which had no effect on proliferation in two experiments. The α2As guanabenz and prazosin increased NSC neuronal differentiation in two experiments, but no data was collected on the effects of α2As on viability. There were mixed results regarding the effects of CCB viability, with most (*n* = 5) experiments reporting no effect. However, one experiment each indicated that benidipine decreases and amlodipine increases NSC viability. Conflicting results were reported for nifedipine effects on NSC neuronal differentiation, with two experiments reporting a decrease and one an increase. Only one experiment investigated A2RA, showing that losartan had no effect on NSC proliferation. Propranolol was the only drug investigated from the BB class. Two experiments reported decreased NSC proliferation under the presence of propranolol, and one experiment did not find an effect.

**TABLE 3:** Number of experiments reporting an inhibitory (–), neutral (±), or stimulating (+) effect of drugs on neural stem cell (NSC) characteristics.

		Proliferation			Differentiation			Viability		
	Drug	–	±	+	–	±	+	–	±	+
Alpha-2 adrenoceptor agonists	Guanabenz	2	0	0	0	0	1	0	0	0
	Clonidine	3	0	0	0	0	0	0	0	0
	Prazosin	0	2	0	0	0	1	0	0	0
Calcium channel blockers	Amlo-dipine	0	0	0	0	0	0	0	2	1
	Beni-dipine	0	0	0	0	0	0	1	2	0
	Nifedipine	0	0	0	2	0	1	0	1	0
	Nitren-dipine	1	0	0	0	0	0	0	0	0
Angiotensin 2R antagonists	Losartan	0	1	0	0	0	0	0	0	0
Beta blockers	Propranolol	2	1	0	1	0	0	0	0	0
Statins	Lovastatin	0	0	0	0	0	0	13	0	0
	Atorva-statin	0	0	1	0	0	0	0	0	0
	Prava-statin	4	0	2	1	0	0	0	5	0
	Simva-statin	5	1	1	1	0	1	3	2	0

A relatively high number of experiments investigated the effect of statins. Lovastatin decreased NSC viability in 13 experiments. Conflicting results were reported for pravastatin with four experiments reporting a decrease of NSC proliferation but two reporting an increase. Moreover, five experiments reported no effect of pravastatin on NSC viability. Experiments revealed that simvastatin decreases NSC proliferation (*n* = 5 experiments) and viability (*n* = 3 experiments), but one experiment each reported no effect or even an increase in proliferation. Two experiments did not find an effect on NSC viability. Conflicting results were obtained for NSC neuronal differentiation in the presence of simvastatin with one experiment each reporting a decrease and an increase, respectively.

### Risk of bias assessment and reporting quality

The risk of bias assessment results are provided in Supplementary Table 2. The overall reporting of quality was mostly incomplete. Only two out of 21 studies reported blinding, four studies provided exposure classification (i.e., target validation, compound verification), and seven a statement of absence of conflict of interest. Common statistical issues identified in 17 studies were the use of statistical tests that require normally distributed data (e.g., *t*-test or ANOVA) without reporting that normal distribution or the homogeneity of variance was tested or confirmed, a low sample size that was insufficient (*n* = 3–4 per group) to assume a normal distribution, or no adjustments for multiple comparisons.

### Methods and conditions in experiments assessing neural stem cell characteristics

A detailed overview of the methods used in the individual studies/experiments is given in Supplementary Tables 2–5. The tables contain bibliographic information, drug doses applied, origin of NSCs, age of animals when NSCs were derived from animals, cell culture conditions and drug exposure times. The tables also list methods used to assess proliferation (Supplementary Table 3), differentiation (Supplementary Table 4), and viability (Supplementary Table 5), respectively.

### Proliferation

**FIGURE 2:**
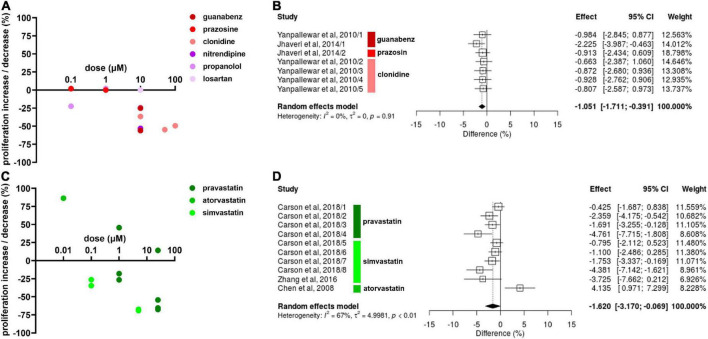
Effect of antihypertensive drugs and statins on neural stem cell (NSC) proliferation. **(A)** Results include effects of α2As (guanabenz, prazosin, and clonidine), calcium channel blockers (CCBs) (nitrendipine), beta blockers (BBs) (propranolol), and angiotensin 2 receptors antagonists (A2RA) (losartan) after 1–14 days exposure at varying doses. **(B)** Forest plot showing the effect sizes of experiments reporting the effect of α2As guanabenz (10 μM), prazosin (1 μM), and clonidine (10, 50, and 100 μM) on NSC proliferation. The overall effect was statistically significant (*p* < 0.01). **(C)** Percentage increase/decrease in NSC proliferation after 3–7 days exposure of varying doses of statins compared to untreated controls. **(D)** A forest plot showing the range in effect sizes of experiments reporting the effect of statins pravastatin (1 and 25 μM) atorvastatin (0.1 μM), and simvastatin (0.1, 5, and 25 μM) on NSC proliferation. There was a statistically significant effect (*p* = 0.041).

The α2As guanabenz (10 μM) and clonidine (10, 50, and 100 μM) decreased NSC proliferation whereas prazosin (0.1 and 1 μM) had no effect on NSC proliferation (Jhaveri et al., 2010; Yanpallewar et al., 2010; Jhaveri et al., 2014; Figure 2A). The CCB nitrendipine (Pincus et al., 1991) and the BB propranolol (Jhaveri et al., 2014) decreased NSC proliferation at 10 μM. Propranolol also decreased NSC proliferation at 0.1 μM in one experiment (Jhaveri et al., 2014) but showed no effect at 1 μM in another experiment (Jhaveri et al., 2010). The A2RA losartan had no effect on NSC proliferation at 10 μM, but NSCs were only exposed to losartan for one hour in this study (Chao et al., 2013). Due to insufficient data on other antihypertensive drug classes, only α2As were included in the meta-analysis for its effect on NSC proliferation (Figure 2B). The meta-analysis revealed that clonidine, prazosin and guanabenz decrease NSC proliferation by a significant, albeit small magnitude (Hedges’ g SMD, −1.05, 95% CI −1.71 to −0.039, *p* = 0.0018).

Pravastatin had varying effects on NSCs depending on the dose and time of exposure (Figure 2C; Carson et al., 2018). At 1 μM, pravastatin increased NSC proliferation by 45.7% after 1 day, but decreased NSC proliferation by 17.9% after 3 days and by 26.5% after 5 days of exposure. Similar results were reported for 25 μM, with a 14.2% increase after 1 day of exposure, 54.3% decrease after 3 days and 67.5% decrease after 5 days of exposure. Simvastatin decreased NSC proliferation at all doses, with 34.5 and 26.3% decreases at 0.1 μM, 69.0 and 67.4% decrease at 5 μM (Carson et al., 2018), and a 65.5% decrease at 25 μM at 2–5 days of exposure (Zhang et al., 2016). Despite the varying results, the effect of pravastatin and simvastatin reducing NSC proliferation were statistically significant according to the meta-analysis (Hedges’ g SMD, −1.62, 95% CI −3.17 to –0.069, *p* = 0.041; Figure 2D).

### Neuronal differentiation

**FIGURE 3:**
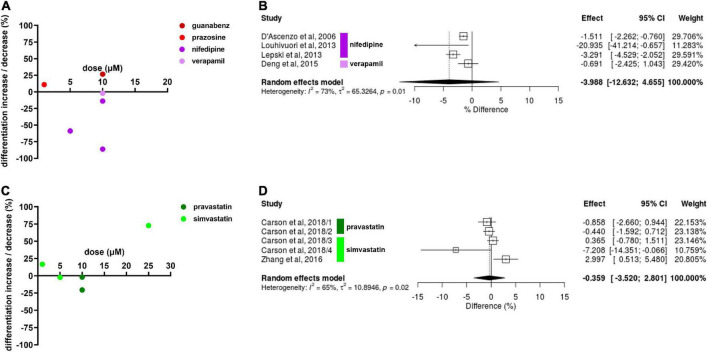
Drug effects on neural stem cell (NSC) neuronal differentiation. **(A)** The effect of calcium channel blockers (CCBs) and α2As on NSC differentiation. Percentage increase/decrease in NSC proliferation after exposure to varying doses compared to untreated controls. Note that the time of exposure differed from 5 to 14 days. **(B)** Forest plot showing the effect sizes of experiments reporting the effect of CCBs on NSC differentiation. No statistical significance was reached (*p* = 0.37). **(C)** Effects of simvastatin and pravastatin on NSC differentiation after 3–6 days exposure. **(D)** Forest plot showing the effect sizes of reported from experiments investigating potential effects of simvastatin (1, 10, and 25 μM) and pravastatin (10 μM) on NSC neuronal differentiation. No statistically significant effect was revealed (*p* = 0.82).

We focused on neuronal differentiation for best comparability between results. Diverse results were reported for drug effects on NSC neuronal differentiation. Nifedipine, a CCB, decreased NSC differentiation by 86.1% at 10 μM in one experiment (Louhivuori et al., 2013), and decreased differentiation by 13.9% in another at the same concentration. Drug exposure in the latter experiment was 2 days longer (7 vs. 5 days). Nifedipine decreased NSC differentiation by 58.8% at 5 μM (D’Ascenzo et al., 2006). Verapamil, another CCB, decreased NSC differentiation by 2% at 10 μM after 7 days of exposure (Deng et al., 2015). Guanabenz (10 μM), and prazosin (1 μM) increased NSC differentiation by 26.7 and 10.9%, respectively, after 14 days of exposure (Jhaveri et al., 2014). One experiment revealed propranolol decreases NSC differentiation by 22.6% at 10 μM after 14 days of drug exposure (Figure 3A). Only the data on CCBs was sufficient for meta-analysis. CCBs showed to inhibit NSC differentiation, however, this effect was not statistically significant (Hedges’ g SMD, −3.99, 95% CI −12.6 to 4.66, *p* = 0.37; Figure 3B).

Simvastatin decreased NSC differentiation by 2.2% at 5 μM but increased by 16.7% at 1 μM in one experiment (Carson et al., 2018). The latter result was confirmed by another experiment in a different study, revealing a 72.7% increase of NSC differentiation at 25 μM (Zhang et al., 2016). Pravastatin decreased NSC differentiation by 2% and in another experiment by 20.6% at 10 μM (Carson et al., 2018; Figure 3C). The diversity in the effect sizes produced from this data was high so statistical significance was not reached (Hedges’ g SMD, −0.36, 95% CI −3.52 to 2.80, *p* = 0.82; Figure 3D).

### Viability

**FIGURE 4:**
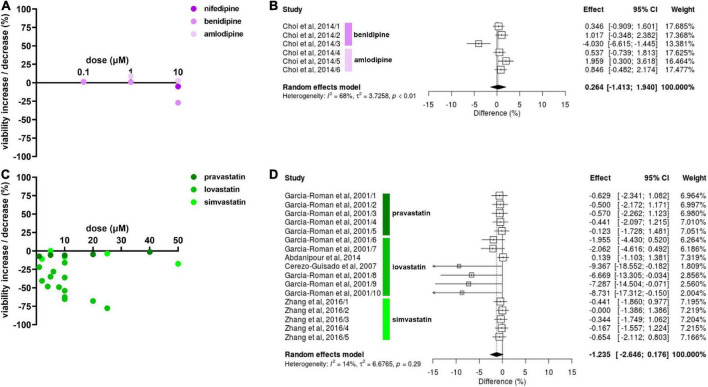
Drug effects on neural stem cell (NSC) viability. **(A)** The effect of calcium channel blockers (CCBs) amlodipine and benidipine on NSC viability after 24 h of exposure. **(B)** Forest plot showing the effect sizes of experiments on amlodipine (0.1, 1, and 10 μM) and benidipine (0.1, 1, and 10 μM). There were no statistically significant effects of antihypertensives on NSC viability (*p* = 0.76). **(C)** Effects of simvastatin and pravastatin on NSC viability after 24–48 h exposure. **(D)** Forest plot showing the effect sizes of experiments reporting the effect of lovastatin (1, 5, 6, 10, 20, and 40 μM), simvastatin (2, 5, 10, 25, and 50 μM), and pravastatin (2, 5, 10, 25, and 50 μM). Although there was a statistically significant effect of lovastatin on reducing NSC viability (*p* = 0.02), no statistically significant overall effect on NSC viability was detected (*p* = 0.086).

Effects of antihypertensive drugs on NSC viability were only reported for CCBs. Most experiments showed no or only minimal effects but benidipine decreased NSC viability by 27.0% at 10 μM (Choi et al., 2014; Figure 4A). Amlodipine slightly increased NSC viability at 1 μM (9.0%) and 10 μM (3.0%) (Choi et al., 2014) whereas nifedipine slightly decreased viability by 5.0% at 1 μM (Kim et al., 2018). CCBs had no effect on NSC viability at 0.1 μM. The meta-analysis did not reveal any statistically significant effect of CCBs on NSC viability (Hedges’ g SMD, 0.26, 95% CI −1.41 to 1.94, *p* = 0.76; Figure 4B).

Lovastatin decreased NSC viability in 13 experiments, reported in four publications, and the NSC viability reduction by lovastatin increased with dose. The highest decrease (77.6%) was reported at 40 μM (García-Román et al., 2001; Figure 4C). Interestingly, pravastatin and simvastatin had no effect on NSC viability even at high doses of up to 50 μM while 2 μM of lovastatin reduced NSC viability by 22.0% compared to pravastatin at −7.2% (García-Román et al., 2001). In summary, lovastatin reduced NSC viability (Hedges’ g SMD, −2.69, 95% CI −4.96 to −0.42, *p* = 0.02; Figure 4D) but there was no relevant effect for either pravastatin or simvastatin. Thus, no overall statin effect on viability was found (Hedges’ g SMD, −1.24, 95% CI −2.65 to 0.18, *p* = 0.086; Figure 4D).

### Migration

Drug effects on NSC migration were only investigated in three experiments reported in two publications. Clonidine reduced NSC migration by 17.2% at 1 μM (over 2 days of exposure) and 8.0% at 10 μM (over 1 day) (Hiramoto et al., 2008) while simvastatin increased NSC migration by 187.0% at 6 μM (Zhang et al., 2016) (data not shown). The data on NSC migration was not sufficient to conduct a meta-analysis and it is therefore difficult to draw meaningful conclusions from these findings.

## Discussion

**FIGURE 5:**
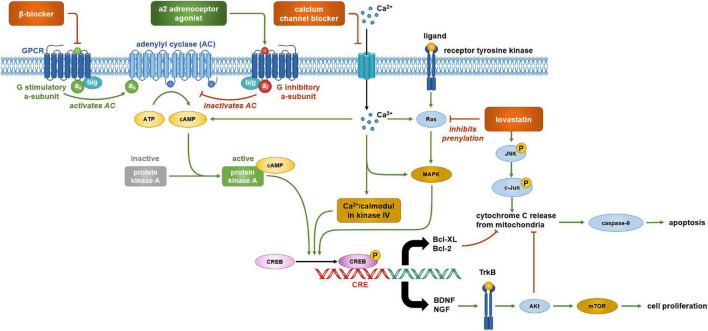
Cell signaling pathways that are potentially involved in regulating neural stem cell (NSC) viability and proliferation. α2 adrenergic receptors are coupled with an inhibitory *G* protein (*G*_*i*_) and β-adrenergic receptors are coupled with stimulatory *G* protein (*G*_*s*_). Activation of α2 adrenergic receptors by agonists guanabenz, clonidine, or prazosin reduces cyclic AMP (cAMP) formation and cAMP response element-binding protein (CREB) phosphorylation. Propranolol has a similar effect by blocking the stimulatory action of the G_s_α subunit. CREB phosphorylation activates the transcription of brain derived neurotrophic factor (BDNF) and nerve growth factor (NGF) which promote cell proliferation by binding to tyrosine receptor kinase B (TrkB) activating the mTOR complex *via* Akt. Hence, lowering CREB phosphorylation by α2 adrenergic receptors agonists and β-adrenergic receptors antagonists may reduce NSC proliferation. cAMP response element-binding protein phosphorylation is also important in the transcription of key genes involved in cell survival such as the anti-apoptotic proteins Bcl-2 and Bcl-XL, and hence, may be targets of lovastatin treatment. Lovastatin inhibits the prenylation of Ras which plays a role in CREB phosphorylation by activating MAPK. This reduction in CREB phosphorylation decreases Bcl-2 and Bcl-XL expression, upregulating apoptosis. Lovastatin also activates c-Jun N terminal kinases (JNK) which further upregulate apoptosis *via* the activation of pro-apoptotic proteins that stimulate the release of cytochrome c from mitochondria. Cytochrome c activates caspase-9 which initiates apoptosis. Akt, activated by growth factor binding to TrkB, increases cell viability by inhibiting pro-apoptotic proteins, preventing the activation of caspase-9 and apoptosis. Therefore, a reduction in BDNF and NGF gene expression by lovastatin reducing the inhibition of apoptosis may be the underlying mechanism explaining the decreased NSC viability after treatment. An influx of calcium into a cell is important in this signaling cascade as it further stimulates cAMP formation and CREB phosphorylation by calmodulin kinase IV. Blocking this influx of calcium using an L-type calcium channel blocker, such as nifedipine, would reduce these actions. As a result, L-type calcium channel blockers may reduce cell viability by the reduction of CREB phosphorylation, decreasing levels of anti-apoptotic protein Bcl-2 as well as the growth factors BDNF and NGF.

Our findings provide support for the hypothesis that antihypertensive drugs and statins exert effects on NSC characteristics. However, the evidence is preliminary and sparse as a widespread and systematic analysis of such effects is still lacking, and some results were conflicting. Nevertheless, the data aids to derive working hypotheses that may be investigated systematically in subsequent studies. Moreover, it is possible to derive further hypotheses on potential mechanisms behind the observed effects (Figure 5).

### Lovastatin may affect neural stem cell viability by decreasing the expression of anti-apoptotic proteins

Lovastatin can affect the intrinsic apoptosis pathway which involves the interaction of pro- and anti-apoptotic proteins (García-Román et al., 2001; Buggins and Pepper, 2010). Lovastatin at 10 μM was found to decrease the levels of the anti-apoptotic proteins Bcl-2 and Bcl-XL (García-Román et al., 2001), disbalancing the pathway toward apoptotic signaling which could explain the lovastatin-associated decrease of NSC viability. Pravastatin does not influence the expression of these anti-apoptotic proteins (Carson et al., 2018), explaining why it had little impact on NSC viability. Lovastatin also increases the expression of the pro-apoptotic protein BimEL at 10 μM after 12 h of exposure (Cerezo-Guisado et al., 2007). Lovastatin further activates Jun N-terminal kinases (JNKs) (García-Román et al., 2001). JNKs stimulate apoptosis by trans-activating c-Jun which increases the expression of pro-apoptotic genes including Bim. JNKs further phosphorylate Bcl-2 and Bcl-XL, inhibiting their anti-apoptotic properties (Dhanasekaran and Reddy, 2008). Bcl-2 and Bcl-XL inhibit pro-apoptotic proteins that stimulate cytochrome c release from mitochondria. Cytochrome c release activates the enzyme caspase-9 which initiates apoptosis (Brentnall et al., 2013). These findings provide strong support for the hypothesis that lovastatin can reduce NSC viability, and similar mechanisms may exist for other drugs.

### Statins may block protein prenylation

Prenylation enables the anchoring of proteins to the cell membrane by lipid modification of target proteins such as Ras (Kennedy et al., 2003). Ras induces cell signaling pathways including the JNK-MAP kinase pathway which plays a role in apoptosis by mediating the expression of pro- and anti-apoptotic proteins as mentioned previously (Kennedy et al., 2003; Dhanasekaran and Reddy, 2008). Isoprenoids such as geranylgeranyl, pyrophosphate, and farnesyl pyrophosphate play a major role in prenylation (Casey and Seabra, 1996) and are formed *via* the cholesterol biosynthesis pathway subsequently to the formation of mevalonate. Statins prevent mevalonate synthesis by inhibiting 3-hydroxy-3-methylglutaryl coenzyme A reductase, reducing isoprenoid production, and thus protein prenylation. Consequently, lovastatin inhibits Ras prenylation. This suggests a mechanism by which lovastatin reduces NSC viability *via* impaired Ras-mediated signal transduction (García-Román et al., 2001). Effects on isoprenoid production may also explain other simvastatin effects reported in the literature. For instance, simvastatin increases Wnt signaling, and thus neuronal differentiation by reducing isoprenoid production (Casey and Seabra, 1996), but the exact mechanisms behind this effect need further exploration.

However, there may be other pathways involved in simvastatin effects that may also help to explain the diverse effects observed on NSC neuronal differentiation. One experiment reported that simvastatin reduces NSC neuronal differentiation by a mechanism independent of the cholesterol biosynthesis pathway, as mevalonate supplementation did not rescue NSC neuronal differentiation (Carson et al., 2018). Another study found that simvastatin increased NSC neuronal differentiation *via* reduced prenylation, with the exact mechanism of this being unknown (Zhang et al., 2016). It is clear that protein prenylation is important in pathways that regulate NSC viability and differentiation, and that statins reduce prenylation by inhibiting isoprenoid production, however, the link between these is not well understood and proposes an area for future research aiming to identify the full mechanism behind the impact of statins on NSCs.

### The effects of nifedipine on neural stem cell neuronal differentiation and a potential role of cyclic AMP

The meta-analysis of CCBs on NSC neuronal differentiation did not reveal significant effects but this may be due to the small number of experiments and the moderate effect sizes being observed. Interestingly, nifedipine inhibited NSC neuronal differentiation in three separate publications. This may suggest a role for voltage gated L-type calcium ion channels in NSC neuronal differentiation and maturation. Indeed, developing neurons express L-type calcium channels and calcium influxes aid neuronal differentiation (D’Ascenzo et al., 2006). One potential role of L-type calcium ion channels is to ensure the integration of newly differentiated neurons into established neural circuits. This may explain why treatment with nifedipine, which blocks this ion channel, inhibits NSC neuronal differentiation (West et al., 2001; Giachino et al., 2005; Lepski et al., 2013).

Small Ca^2+^ currents can be detected early in NSC neuronal differentiation and are believed to stimulate this differentiation (Giachino et al., 2005). This effect may be related to the activation of calcium-sensitive adenylate cyclase (West et al., 2001). Adenylate cyclase catalyzes the conversion of ATP to cyclic AMP (cAMP). Activation of cAMP is important for the phosphorylation of the cAMP response element-binding protein (CREB). CREB is a transcription factor which binds to the cAMP response element (CRE) upon phosphorylation and regulates the transcription of genes involved in cell development (West et al., 2001). Phosphorylated CREB (pCREB) is upregulated in differentiating NSCs and dictates neuronal morphology. Cell exposure to nifedipine reduces pCREB levels and thus differentiation (Lepski et al., 2013). Moreover, CREB knockout mice show impaired neuroblast viability and reduced NSC neuronal differentiation (Giachino et al., 2005). Besides cAMP, Ca^2+^ influx can also regulate pCREB *via* calmodulin kinase IV, MAP kinase, and Ras.

### Cyclic AMP and neural stem cell proliferation

α2 and β-adrenergic receptors are G-protein-coupled receptors. In particular, α2 adrenergic receptors couple with the inhibitory *G* protein (*G*_*i*_) which inhibits the action of adenylate cyclase and reduces cAMP levels. In turn, β-adrenergic receptors couple with the stimulatory *G* protein (*G*_*s*_) which stimulates adenylate cyclase and increases cAMP levels (Hein and Kobilka, 1995). β-adrenergic receptor antagonists such as propranolol and the α2-adrenergic receptor agonists guanabenz and clonidine (but not prazosin) reduced NSC proliferation. This may be due to the knock-on effect on cAMP *via* the *G*-protein coupled receptors. Inhibiting β-adrenergic receptors by propranolol may impede cAMP production by blocking the action of adenylate cyclase, whereas activating α2 adrenergic receptors would also inhibit cAMP production *via* the release of *G*_*i*_ that blocks adenylate cyclase. These actions would lead to a reduction of CREB phosphorylation. Indeed, pCREB is not only important for NSC neuronal differentiation, but plays a role in NSC viability and proliferation (West et al., 2001).

Phosphorylated CREB may affect NSC viability by increasing the expression of the anti-apoptotic proteins Bcl-2 and Bcl-XL. This is mediated by the CREB signaling pathway and induces various neurotrophins such as brain derived neurotrophic factor (BDNF) and nerve growth factor (NGF) (Riccio et al., 1999; West et al., 2001). BDNF binds to tyrosine receptor kinase B (TrkB) which upregulates cell proliferation by activating the mTOR complex (Sato et al., 2010; Bath et al., 2012). Further, growth factor binding to TrkB activates Akt which inhibits pro-apoptotic protein BAD and thus apoptosis (Downward, 2004). The effect of drugs influencing the Ras/cAMP-CREB pathway and their relationship with growth factors such as BDNF proposes an area for future research as there are potential links between the two that may explain the effects on NSC proliferation and viability.

### *In vivo* effects of drugs on neural stem cell characteristics

We only included *in vitro* experiments in our systematic review and meta-analysis for better comparability of the results. There are *in vivo* studies reporting effects of anti-hypertensives and anti-dyslipidemics on NSCs, but these studies are relatively rare and almost exclusively focus on a disease condition. For instance, the effects of atorvastatin on endogenous neuronal progenitor cell proliferation have been investigated in stroke (Chen et al., 2008), and data from the *in vitro* part of this paper are included in our data set. Since stroke itself increases endogenous NSC proliferation, it is unclear how much of the observed effects can be attributed to atorvastatin. Other studies used anti-hypertensives or anti-dyslipidemics as co-treatments to another approach such as adult stem cell transplantation (for instance Cui et al., 2012). This makes it very hard to discriminate the effects of the drug from those of both the co-treatment and the disease condition.

The effects of drugs on NSC characteristics *in vivo* may differ from those *in vitro* as aspects including pharmacodynamics, pharmacokinetics or the blood-brain barrier may affect drug doses in neurogenic areas within the brain as well as exposure times. Thus, there is a need for *in vivo* studies specifically designed to investigate drug effects on NSC characteristics, ideally both in the absence and presence of a particular disease condition.

To the best of our knowledge, no clinical trial investigating NSCs for neurodegenerative diseases has so far considered effects of anti-hypertensives or anti-dyslipidemics. If results obtained by future research confirm the hypothesis being derived from our research, drug effects on endogenous but also therapeutically administered NSCs will be of relevance for the design of clinical trials as well as the interpretation of their results.

## Limitations

Although our results allow the derivation of working hypotheses, our approach has some limitations. Most importantly, the overall number of experiments retrieved from the literature was relatively small and individual experiments were often different with respect to drug dose and exposure time, introducing considerable heterogeneity. This is not surprising as drug effects on NSC characteristics were often reported as side findings. Thorough downstream research specifically designed to investigate these effects and the underlying mechanisms, avoiding heterogeneity in study design and thereby increasing validity, is therefore required.

Second, all experiments analyzed were conducted *in vitro.* This allows better standardization of procedures and more direct investigations on a cellular level. However, it would be interesting to see whether similar results are observed *in vivo*.

Third, exposure times to different drugs varied throughout the studies which may have included some form of bias. Future experiments will have to standardize exposure times for consistent results. Finally, there were considerable differences regarding the methods by which potential drug effects were investigated, again calling for more standardized protocols in future research. This also applies for future *in vivo* studies.

Fourth, the overall reporting quality and sample sizes in the included studies was often low. Future studies should report important methodological details and apply respective actions to reduce the overall risk of bias. Studies should also be adequately powered.

## Conclusion

Here, we present support for the hypothesis that commonly applied drugs, statins, and antihypertensives, may affect key NSC characteristics. We performed a systematic review and meta-analysis on the available literature to collect preliminary evidence for such effects, occasionally reported as side findings in original research. We also present potential mechanisms by which these effects may be mediated. We finally identified key points to be addressed in future research to thoroughly test this hypothesis and to answer open question which are important for the translation of experimental NSC treatments of neurodegenerative diseases into clinical practice.

## Data availability statement

Publicly available datasets were analyzed in this study. The original data are available from the corresponding author upon reasonable request.

## Author contributions

KRHM and HV-B: performing the systematic research and the data extraction. KRHM and JB: meta-analysis and drafting the manuscript. MZ, ND, and JB: conceptualizing the research and editing the manuscript. ND and JB: the data extraction. KRHM, ND, and JB: data extraction. MZ: risk of biases analysis. All authors contributed to the article and agreed on the final version of the manuscript.

## Footnotes

1
https://camarades.shinyapps.io/meta-analysis-app/

## References

[B1] Bath K. G. Akins M. R. Lee F. S. (2012). BDNF control of adult SVZ neurogenesis. *Dev. Psychobiol.* 54 578–589. 10.1002/dev.20546 21432850 PMC3139728

[B2] Boltze J. Arnold A. Walczak P. Jolkkonen J. Cui L. Wagner D.-C. (2015). The dark side of the force - constraints and complications of cell therapies for stroke. *Front. Neurol.* 6:155. 10.3389/fneur.2015.00155 26257702 PMC4507146

[B3] Brentnall M. Rodriguez-Menocal L. Ladron De Guevara R. Cepero E. Boise L. H. (2013). Caspase-9, caspase-3 and caspase-7 have distinct roles during intrinsic apoptosis. *BMC Cell. Biol.* 14:32. 10.1186/1471-2121-14-32 23834359 PMC3710246

[B4] Buggins A. G. S. Pepper C. J. (2010). The role of Bcl-2 family proteins in chronic lymphocytic leukaemia. *Leuk. Res.* 34 837–842. 10.1016/j.leukres.2010.03.011 20359747

[B5] Carson R. A. Rudine A. C. Tally S. J. Franks A. L. Frahm K. A. Waldman J. K. (2018). Statins impact primary embryonic mouse neural stem cell survival, cell death, and fate through distinct mechanisms. *PLoS One* 13:e0196387. 10.1371/journal.pone.0196387 29738536 PMC5940229

[B6] Casey P. J. Seabra M. C. (1996). Protein prenyltransferases. *J. Biol. Chem.* 271 5289–5292. 10.1074/jbc.271.10.5289 8621375

[B7] Cerezo-Guisado M. I. Alvarez-Barrientos A. Argent R. García-Marín L. J. Bragado M. J. Lorenzo M. J. (2007). c-Jun N-terminal protein kinase signalling pathway mediates lovastatin-induced rat brain neuroblast apoptosis. *Biochim. Biophys. Acta* 1771 164–176. 10.1016/j.bbalip.2006.12.002 17251057

[B8] Chao J. Yang L. Buch S. Gao L. (2013). Angiotensin II increased neuronal stem cell proliferation: Role of AT2R. *PLoS One* 8:e63488. 10.1371/journal.pone.0063488 23691054 PMC3655161

[B9] Chen J. Zacharek A. Li A. Cui X. Roberts C. Lu M. (2008). Atorvastatin promotes presenilin-1 expression and Notch1 activity and increases neural progenitor cell proliferation after stroke. *Stroke* 39 220–226. 10.1161/STROKEAHA.107.490946 18063826 PMC2792764

[B10] Choi N.-Y. Choi H. Park H.-H. Lee E.-H. Yu H.-J. Lee K. Y. (2014). Neuroprotective effects of amlodipine besylate and benidipine hydrochloride on oxidative stress-injured neural stem cells. *Brain Res.* 1551 1–12. 10.1016/j.brainres.2014.01.016 24440775

[B11] Cui X. Chopp M. Shehadah A. Zacharek A. Kuzmin-Nichols N. Sanberg C. D. (2012). Therapeutic benefit of treatment of stroke with simvastatin and human umbilical cord blood cells: Neurogenesis, synaptic plasticity, and axon growth. *Cell Transplant.* 21 845–856. 10.3727/096368911X627417 22405262 PMC3442771

[B12] D’Ascenzo M. Piacentini R. Casalbore P. Budoni M. Pallini R. Azzena G. B. (2006). Role of L-type Ca2+ channels in neural stem/progenitor cell differentiation. *Eur. J. Neurosci.* 23 935–944. 10.1111/j.1460-9568.2006.04628.x 16519658

[B13] Deng S. Hou G. Xue Z. Zhang L. Zhou Y. Liu C. (2015). Vitamin E isomer δ-tocopherol enhances the efficiency of neural stem cell differentiation via L-type calcium channel. *Neurosci. Lett.* 585 166–170. 10.1016/j.neulet.2014.11.031 25445352

[B14] Dhanasekaran D. N. Reddy E. P. (2008). JNK signaling in apoptosis. *Oncogene* 27 6245–6251. 10.1038/onc.2008.301 18931691 PMC3063296

[B15] Downward J. (2004). PI 3-kinase, Akt and cell survival. Semin. *Cell. Dev. Biol.* 15 177–182. 10.1016/j.semcdb.2004.01.002 15209377

[B16] García-Román N. Alvarez A. M. Toro M. J. Montes A. Lorenzo M. J. (2001). Lovastatin induces apoptosis of spontaneously immortalized rat brain neuroblasts: Involvement of nonsterol isoprenoid biosynthesis inhibition. *Mol. Cell. Neurosci.* 17 329–341. 10.1006/mcne.2000.0904 11178870

[B17] Giachino C. De Marchis S. Giampietro C. Parlato R. Perroteau I. Schütz G. (2005). cAMP response element-binding protein regulates differentiation and survival of newborn neurons in the olfactory bulb. *J. Neurosci.* 25 10105–10118. 10.1523/JNEUROSCI.3512-05.2005 16267218 PMC6725785

[B18] Hein L. Kobilka B. K. (1995). Adrenergic receptor signal transduction and regulation. *Neuropharmacology* 34 357–366. 10.1016/0028-3908(95)00018-2 7566466

[B19] Higgins J. P. T. Thomas J. Chandler J. Cumpston M. Li T. Page M. J. (eds) (2022). *Cochrane handbook for systematic reviews of interventions version 6.3 (updated February 2022).* London, UK: Cochrane.

[B20] Hiramoto T. Satoh Y. Takishima K. Watanabe Y. (2008). Induction of cell migration of neural progenitor cells in vitro by alpha-1 adrenergic receptor and dopamine D1 receptor stimulation. *Neuroreport* 19 793–797. 10.1097/WNR.0b013e3282fd1270 18446092

[B21] Ikhsan M. Palumbo A. Rose D. Zille M. Boltze J. (2019). Neuronal stem cell and drug interactions: A systematic review and meta-analysis: Concise review. *Stem Cells Transl. Med.* 8 1202–1211. 10.1002/sctm.19-0020 31313515 PMC6811698

[B22] Janowski M. Wagner D.-C. Boltze J. (2015). Stem cell-based tissue replacement after stroke: factual necessity or notorious fiction? *Stroke* 46 2354–2363. 10.1161/STROKEAHA.114.007803 26106118 PMC4519410

[B23] Jhaveri D. J. Mackay E. W. Hamlin A. S. Marathe S. V. Nandam L. S. Vaidya V. A. (2010). Norepinephrine directly activates adult hippocampal precursors via beta3-adrenergic receptors. *J. Neurosci.* 30 2795–2806. 10.1523/JNEUROSCI.3780-09.2010 20164362 PMC2837927

[B24] Jhaveri D. J. Nanavaty I. Prosper B. W. Marathe S. Husain B. F. A. Kernie S. G. (2014). Opposing effects of α2- and β-adrenergic receptor stimulation on quiescent neural precursor cell activity and adult hippocampal neurogenesis. *PLoS One* 9:e98736. 10.1371/journal.pone.0098736 24922313 PMC4055446

[B25] Kalladka D. Sinden J. Pollock K. Haig C. McLean J. Smith W. (2016). Human neural stem cells in patients with chronic ischaemic stroke (PISCES): A phase 1, first-in-man study. *Lancet* 388 787–796. 10.1016/S0140-6736(16)30513-X 27497862

[B26] Kennedy N. J. Sluss H. K. Jones S. N. Bar-Sagi D. Flavell R. A. Davis R. J. (2003). Suppression of Ras-stimulated transformation by the JNK signal transduction pathway. *Genes Dev.* 17 629–637. 10.1101/gad.1062903 12629045 PMC196007

[B27] Kim J.-W. Oh H. A. Lee S. H. Kim K. C. Eun P. H. Ko M. J. (2018). T-Type calcium channels are required to maintain viability of neural progenitor cells. *Biomol. Ther.* 26 439–445. 10.4062/biomolther.2017.223 29463073 PMC6131011

[B28] Leal-Galicia P. Chávez-Hernández M. E. Mata F. Mata-Luévanos J. Rodríguez-Serrano L. M. Tapia-de-Jesús A. (2021). Adult neurogenesis: A story ranging from controversial new neurogenic areas and human adult neurogenesis to molecular regulation. *Int. J. Mol. Sci.* 22:11489. 10.3390/ijms222111489 34768919 PMC8584254

[B29] Lepski G. Jannes C. E. Nikkhah G. Bischofberger J. (2013). cAMP promotes the differentiation of neural progenitor cells *in vitro via* modulation of voltage-gated calcium channels. *Front. Cell. Neurosci.* 7:155. 10.3389/fncel.2013.00155 24065885 PMC3777016

[B30] Limke T. L. Rao M. S. (2002). Neural stem cells in aging and disease. *J. Cell. Mol. Med.* 6 475–496. 10.1111/j.1582-4934.2002.tb00451.x 12611637 PMC6741307

[B31] Louhivuori L. M. Louhivuori V. Wigren H.-K. Hakala E. Jansson L. C. Nordström T. (2013). Role of low voltage activated calcium channels in neuritogenesis and active migration of embryonic neural progenitor cells. *Stem Cells Dev.* 22 1206–1219. 10.1089/scd.2012.0234 23234460

[B32] Mine Y. Tatarishvili J. Oki K. Monni E. Kokaia Z. Lindvall O. (2013). Grafted human neural stem cells enhance several steps of endogenous neurogenesis and improve behavioral recovery after middle cerebral artery occlusion in rats. *Neurobiol. Dis.* 52 191–203. 10.1016/j.nbd.2012.12.006 23276704

[B33] Moher D. Liberati A. Tetzlaff J. Altman D. G. Prisma Group. (2009). Preferred reporting items for systematic reviews and meta-analyses: The PRISMA statement. *PLoS Med.* 6:e1000097. 10.1371/journal.pmed.1000097 19621072 PMC2707599

[B34] Moreno-Jiménez E. P. Terreros-Roncal J. Flor-García M. Rábano A. Llorens-Martín M. (2021). Evidences for adult hippocampal neurogenesis in humans. *J. Neurosci.* 41 2541–2553. 10.1523/JNEUROSCI.0675-20.2020 33762406 PMC8018741

[B35] Muir K. W. Bulters D. Willmot M. Sprigg N. Dixit A. Ward N. (2020). Intracerebral implantation of human neural stem cells and motor recovery after stroke: Multicentre prospective single-arm study (PISCES-2). *J. Neurol. Neurosurg. Psychiatr.* 91 396–401. 10.1136/jnnp-2019-322515 32041820 PMC7147186

[B36] National Health Service United Kingdom (2018). *Statins.* Available online at: https://www.nhs.uk/conditions/statins/ (accessed on May 15, 2022).

[B37] National Institute for Health and Care Excellence guidelines (2019). *Hypertension in adults: Diagnosis and management.* Available online at: https://www.nice.org.uk/guidance/ng136/chapter/Recommendations#treating-and-monitoring-hypertension (accessed on May 15, 2022). 31577399

[B38] Office of Health Assessment and Translation (2019). *OHAT risk of bias tool for human and animal studies (updated March 2019).* Available online at: https://ntp.niehs.nih.gov/pubhealth/hat/review/index-2.html (accessed on Aug 26, 2022).

[B39] Pincus D. W. DiCicco-Bloom E. Black I. B. (1991). Role of voltage-sensitive calcium channels in mitogenic stimulation of neuroblasts. *Brain Res.* 553 211–214. 10.1016/0006-8993(91)90827-I 1718543

[B40] Riccio A. Ahn S. Davenport C. M. Blendy J. A. Ginty D. D. (1999). Mediation by a CREB family transcription factor of NGF-dependent survival of sympathetic neurons. *Science* 286 2358–2361. 10.1126/science.286.5448.2358 10600750

[B41] Rosenblum S. Wang N. Smith T. N. Pendharkar A. V. Chua J. Y. Birk H. (2012). Timing of intra-arterial neural stem cell transplantation after hypoxia-ischemia influences cell engraftment, survival, and differentiation. *Stroke* 43 1624–1631. 10.1161/STROKEAHA.111.637884 22535265

[B42] Sato A. Sunayama J. Matsuda K. Tachibana K. Sakurada K. Tomiyama A. (2010). Regulation of neural stem/progenitor cell maintenance by PI3K and mTOR. *Neurosci. Lett.* 470 115–120. 10.1016/j.neulet.2009.12.067 20045038

[B43] West A. E. Chen W. G. Dalva M. B. Dolmetsch R. E. Kornhauser J. M. Shaywitz A. J. (2001). Calcium regulation of neuronal gene expression. *Proc. Natl. Acad. Sci. U. S. A.* 98 11024–11031. 10.1073/pnas.191352298 11572963 PMC58677

[B44] Yanpallewar S. U. Fernandes K. Marathe S. V. Vadodaria K. C. Jhaveri D. Rommelfanger K. (2010). Alpha2-adrenoceptor blockade accelerates the neurogenic, neurotrophic, and behavioral effects of chronic antidepressant treatment. *J. Neurosci.* 30 1096–1109. 10.1523/JNEUROSCI.2309-09.2010 20089918 PMC2880491

[B45] Yoon Y. Kim H. S. Jeon I. Noh J.-E. Park H. J. Lee S. (2020). Implantation of the clinical-grade human neural stem cell line, CTX0E03, rescues the behavioral and pathological deficits in the quinolinic acid-lesioned rodent model of Huntington’s disease. *Stem Cells* 38 936–947. 10.1002/stem.3191 32374064 PMC7496241

[B46] Zhang C. Wu J.-M. Liao M. Wang J.-L. Xu C.-J. (2016). The ROCK/GGTase pathway are essential to the proliferation and differentiation of neural stem cells mediated by simvastatin. *J. Mol. Neurosci.* 60 474–485. 10.1007/s12031-016-0811-y 27541019

[B47] Zhang G. Li Y. Reuss J. L. Liu N. Wu C. Li J. (2019). Stable intracerebral transplantation of neural stem cells for the treatment of paralysis due to ischemic stroke. *Stem Cells Transl. Med.* 8 999–1007. 10.1002/sctm.18-0220 31241246 PMC6766600

