# Supplementary material for: Potential effects of commonly applied drugs on neural stem cell proliferation and viability: A hypothesis-generating systematic review and meta-analysis
Source: Front Mol Neurosci. 2022 Oct 5;15:975697. doi: 10.3389/fnmol.2022.975697 (PMC9581168; doi:10.3389/fnmol.2022.975697)
Supplement: Supplementary file 1 [file Data_Sheet_1.PDF]

Supplementary materials

Supplementary table 1: Search strategy

Drugs	NSC	NSC characteristics
1. Angiotensin 2 receptor antagonist<br>2. Azilsartan<br>3. Candesartan<br>4. Losartan<br>5. Eprosartan<br>6. Fimasartan<br>7. #1 OR #2 OR #3 OR #4 OR #5 OR #6<br>8. Telmisartan<br>9. Olmesartan<br>10. Irbesartan<br>11. Valsartan<br>12. #8 OR #9 OR #10 OR #11<br>13. Calcium channel blockers<br>14. Amlodipine<br>15. Diltiazem<br>16. Felodipine<br>17. Verapamil<br>18. Cilnidipine<br>19. #13 OR #14 OR #15 OR #16 OR #17 OR #18<br>20. Clevidipine<br>21. Isradipine<br>22. Lercanidipine<br>23. Levamlodipine<br>24. Nifedipine<br>25. Nimodipine<br>26. #20 OR #21 OR #22 OR #23 OR #25<br>27. ACE inhibitors<br>28. Enalapril<br>29. Fosinopril<br>30. Lisinopril<br>31. Ramipril<br>32. Captopril<br>33. #27 OR #28 OR #29 OR #30 OR #31 OR #32<br>34. Benazepril<br>35. Moexipril<br>36. Perindopril<br>37. Quinapril<br>38. Trandolapril<br>39. #34 OR #35 OR #36 OR #37 OR #38<br>40. Thiazide diuretics<br>41. Thiazide-like diuretics<br>42. Bendroflumethiazide<br>43. Chlorothiazide<br>44. Chlorthalidone<br>45. Indapamide<br>46. Metalozone<br>47. #40 OR #41 OR #42 OR #43 OR #44 OR #45 OR #46<br>48. Hydrochlorothiazide<br>49. Trichlormethiazide<br>50. Polythiazide<br>51. Xipamide<br>52. Clopamide<br>53. #48 OR #49 OR #50 OR #51 OR #52<br>54. Aldosterone receptor inhibitor<br>55. Potassium-sparing diuretic<br>56. Spironolactone<br>57. Eperenone<br>58. Amiloride<br>59. Triamterene<br>60. #54 OR #55 OR #56 OR #57 OR #58 OR #59	103. Neural stem cells<br>104. Neuronal stem cells<br>105. NSCs<br>106. Neural progenitor cells<br>107. Neuronal progenitor cells<br>108. NPCs<br>109. Neural progenitors<br>110. Neuronal progenitors<br>111. Neural precursor cell<br>112. Neuronal precursor cell<br>113. Neural precursors<br>114. Neuronal precursors<br>115. Neural stem cell therapy<br>116. Neuronal stem cell therapy<br>117. Neuroblasts<br>118. #103 OR #104 OR #105 or #106 OR #107 OR #108 OR #109 OR #110 OR #111 OR #112 OR #113 OR #114 OR #115 OR #116 OR #117	119. Neurogenesis<br>120. Differentiation<br>121. Expansion<br>122. Maturation<br>123. Development<br>124. Proliferation<br>125. Amplification<br>126. Self-renewal<br>127. Regeneration<br>128. Division<br>129. Growth<br>130. Migration<br>131. Vitality<br>132. Viability<br>133. Survival<br>134. #119 OR #120 OR #121 or #122 OR #123 OR #124 OR #125 OR #126 OR #127 OR #128 OR #129 OR #130 OR #131 OR #132 OR #133

61. Beta blockers<br>62. Propranolol<br>63. Metoprolol<br>64. Atenolol<br>65. Bisoprolol<br>66. Nadolol<br>67. #61 OR #62 OR #63 OR #64 OR #65 OR #66<br>68. Acebutolol<br>69. Betaxolol<br>70. Carteolol<br>71. Labetolol<br>72. Oxprenolol<br>73. Timolol<br>74. Carvedilol<br>75. #68 OR #69 OR #70 OR #71 OR #72 OR #73 OR #74<br>76. Alpha-1-adrenoceptor antagonists<br>77. Doxazosin<br>78. Prazosin<br>79. Terazosin<br>80. #76 OR #77 OR #78 OR #79<br>81. Alpha-2-adrenoceptor agonists<br>82. Clonidine<br>83. Methyldopa<br>84. Guanabenz<br>85. Guanfacine<br>86. #81 OR #82 OR #83 OR #84 OR #85<br>87. Direct renin inhibitor<br>88. Aliskiren<br>89. #87 OR #88<br>90. Loop diuretics<br>91. Torsemide<br>92. Furosemide<br>93. #90 OR #91 OR #92<br>94. Statins<br>95. Atorvastatin<br>96. Fluvastatin<br>97. Pravastatin<br>98. Rosuvastatin<br>99. Simvastatin<br>100. HMG-CoA<br>101. HMG-CoA reductase inhibitor<br>102. #94 OR #95 OR #96 OR #97 OR #98 OR #99 OR #100 OR #101 OR #102		

Supplemental table 2: Risk of bias assessment of the included studies

			Selection bias	Performance/ detection bias		Attrition bias		Reporting bias		Other bias	
**PMID**	**Author, year**	**Figures**	Randomi-zation	Sample size calculation	Allocation concealment	Blinding of researchers	Exposure classificatio	Complete outcome	Selective reporting	Conflict of interest	Statistical analysis
24440775	Choi et al., 2014	1B, 3B									
29463073	Kim et al., 2018	2F									
11178870	García-Román et al., 2001	1A, 1B									
17251057	Cerezo-Gusado et al., 2006	7C									
31885397	Fakheri et al., 2019	1M									
20089918	Yanpallewar et al., 2010	3F, 3H									
24922313	Jhaveri et al., 2014	1A, 1C, 2C, 3A, 3C, 4B									
20164362	Jhaveri et al., 2010	6A									
23691054	Chao et al., 2013	1B									
1718543	Pincus et al., 1991	3A									
29738536	Carson et al., 2018	1C, S1									
27541019	Zhang et al., 2016	2, 3B									
18063826	Chen et al., 2008	3C									
16519658	D'Ascenzo et al., 2006	7B									
23234460	Louhivuori et al., 2013	8B									
24065885	Lepski et al., 2013	4B									
25445352	Deng et al., 2015	3E									
30073508	Choi et al., 2019	1D									
24390568	Abdanipour et al, 2014	3A									
16952276	Cerezo-Gusado et al., 2007	5A									
18446092	Hiramoto et al, 2008	1A									

The studies were assessed with respect to the effects of commonly applied drugs on neural stem cell proliferation, viability, differentiation, and migration. Bluish green indicates low risk, vermillon high risk, and yellow unclear risk due to lack of information or uncertainty about the potential for bias. A common statistical issue identified is the use of statistical tests that require

normally distributed data (e.g., t-test or ANOVA) without reporting that normal distribution or the homogeneity of variance was tested or confirmed (yellow label). Incorrect statistical tests (red label) were reported when parametric tests were performed with a sample size (n=3-4 per group) insufficient to assume normal distribution or when adjustment for multiple comparisons was not performed.

Supplementary table 3: Characteristics of studies / experiments investigating NSC proliferation

Author, year	PMID	Drug	Doses (µM)	NSC origin	Age of animal of isolation	Culture medium	Drug exposure time	Proliferation assessment
Carson et al., 2018	29738536	pravastatin	1, 1, 1, 25, 25, 25	fetal mouse cortex	embryonic day 14.5	Neurocult medium with proliferation supplements	24 hours	automated cell count
Carson et al., 2018	29738536	simvastatin	0.1, 0.1, 5, 5	fetal mouse cortex	embryonic day 14.5	Neurocult medium with proliferation supplements	3 days	automated cell count
Chen et al., 2008	18063826	atorvastatin	0.01	rat subventricular zone	12 months	DMEM/F-12 with 2% B27, BFGF, EGF, 0.6% glucose, L-glutamine.	2 days	neurosphere formation
Chao et al., 2013	23691054	losartan	10	fetal rat cortex	embryonic day 18	Neural progenitor basal medium, EGF, BFGF, 2% neural survival factor-1	1 hour	CyQuant assay
Jhaveri et al., 2014	24922313	prazosin	1	neurospheres isolated from C57BL/6J mice	8-12 weeks old	Neurocult NSC basal medium with proliferation supplements, 2% bovine serum, heparin, EGF, BFGF	14 days	neurosphere formation
Jhaveri et al., 2014	24922313	guanabenz	10	neurospheres isolated from C57BL/6J mice	8-12 weeks old	Neurocult NSC basal medium with proliferation supplements, 2% bovine serum, heparin, EGF, BFGF	14 days	neurosphere formation
Jhaveri et al., 2014	24922313	propranolol	0.1, 1, 10	neurospheres isolated from C57BL/6J mice	8-12 weeks old	Neurocult NSC basal medium with proliferation supplements, 2% bovine serum, heparin, EGF, BFGF	14 days	neurosphere formation
Jhaveri et al., 2010	20164362	prazosin	0.1	neurospheres isolated from C57BL/6J mice	8-12 weeks old	Neurocult NSC basal medium with proliferation supplements, 2% bovine serum, heparin, EGF, BFGF	10-13 days	neurosphere formation
Pincus et al., 1991	1718543	nitrendipine	10	superior cervical ganglia from Sprague-Dawley rat embryos	embryonic day 15.5	no information provided in paper	2 days	^{3}H-thymidine assay
Yanpallewar et al., 2010	20089918	clonidine	10, 50, 100	neonatal hippocampi from Wistar rats	7 days old	Neurocult NSC basal medium with proliferation supplements, 2% bovine serum, heparin, EGF, BFGF	24 hours	BrdU assay
Yanpallewar et al., 2010	20089918	guanabenz	10	neonatal hippocampi from Wistar rats	7 days old	Neurocult NSC basal medium with proliferation supplements, 2% bovine serum, heparin, EGF, BFGF	24 hours	BrdU assay
Zhang et al., 2016	27541019	simvastatin	25	rat fetuses	embryonic day 14.5	DMEM/F-12 with 2% B27, N2, L-glutamate, streptomycin, penicillin	2 days	Ki-67 assay

Supplementary table 4: Characteristics of studies / experiments investigating NSC differentiation

Author, year	PMID	Drug	Doses (µM)	NSC origin	Age of animal of isolation	Culture medium	Drug exposure time	Differentiation assessment
Carson et al., 2018	29738536	pravastatin	10	mouse cortical region	embryonic day 14.5	Neurocult medium with proliferation supplements	3 days	beta-3 tubulin
Carson et al., 2018	29738536	simvastatin	10, 25, 25	mouse cortical region	embryonic day 14.5	Neurocult medium with proliferation supplements	3 days	beta-3 tubulin
D'Ascenzo et al., 2006	16519658	nifedipine	5	NSCs isolated from mouse brain cortex	0 days old	DMEM/F-12 with 2% B27, BFGF, EGF, 0.6% glucose, L-glutamine	6-12 days	beta-3 tubulin
Deng et al., 2015	25445352	verapamil	10	neurospheres from neonatal Wistar rats	1 day old	Neurobasal-A medium, 2% B27, BFGF, EGF, L-glutamine, heparin, BDNF	7 days	beta-3 tubulin
Jhaveri et al., 2014	24922313	guanabenz	10	neurospheres isolated from C57BL/6J mice	8-12 weeks old	Neurocult NSC basal medium with proliferation supplements, 2% bovine serum, heparin, EGF, BFGF	14 days	beta-3 tubulin
Jhaveri et al., 2014	24922313	prazosin	1	neurospheres isolated from C57BL/6J mice	8-12 weeks old	Neurocult NSC basal medium with proliferation supplements, 2% bovine serum, heparin, EGF, BFGF	14 days	beta-3 tubulin
Jhaveri et al., 2014	24922313	propranolol	10	neurospheres isolated from C57BL/6J mice	8-12 weeks old	Neurocult NSC basal medium with proliferation supplements, 2% bovine serum, heparin, EGF, BFGF	14 days	beta-3 tubulin
Lepski et al., 2013	24065885	nifedipine	10	Sprague Dawley rat fetus	embryonic day 14	DMEM/F-12 with 2% B27, BFGF, EGF, 0.6% glucose, L-glutamine	7 days	MAP-2
Louhivuori et al., 2013	23234460	nifedipine	10	subventricular zone (lateral ventricles)	embryonic day 14	DMEM/F-12 with 2% B27, BFGF, EGF, 0.6% glucose, L-glutamine	5 days	NeuN
Zhang et al., 2016	27541019	simvastatin	25	Sprague Dawley rat fetus	embryonic day 14.5	DMEM/F-12 with B27, glutamine, heparin, penicillin streptomycin	6 days	beta-3-tubulin

Supplementary table 5: Characteristics of studies / experiments investigating NSC viability

Author, year	PMID	Drug	Doses (µM)	NSC origin	Age of animal of isolation	Culture medium	Drug exposure time	Viability assessment
Abdanipour et al., 2014	24390568	lovastatin	6	Wistar rats (200-300g)	Not specified	DMEM with 15% FBS, penicillin, streptomycin	48 hours	MTT Assay
Cerezo-Guisado et al., 2007	17251057	lovastatin	10	neuroblasts from fetal rat cerebral cortices	embryonic day 17	Ham’s F12 with 10% FCS, streptomycin, and penicillin	24 hours	Crystal violet
Choi et al., 2014	24440775	amlodipine	0.1, 1, 10	Sprague-Dawley rat embryonic brain tissue	embryonic day 12-13	N2 medium and basic fibroblast growth factor	24 hours	MTT Assay
		benidipine	0.1, 1, 10					
Fakheri et al., 2019	31885397	lovastatin	2, 4, 6, 8, 10	neurospheres from hippocampus of neonatal Wistar rats	5-10 days old	DMEM/F-12 with 2% B27, BFGF, EGF, streptomycin, penicillin	24 hours	MTT Assay
García-Román et al., 2001	11178870	lovastatin	1, 5, 10, 10, 20, 40	fetal rat cerebral cortices	embryonic day 18	Hanks’ F12 [sic!] with 10% FCS, streptomycin, and penicillin,	24 hours	MTT Assay
García-Román et al., 2001	11178870	pravastatin	1, 5, 10, 20, 40	fetal rat cerebral cortices	embryonic day 19	Hanks’ F12 [sic!] with 10% FCS, streptomycin, and penicillin	24 hours	MTT Assay
Kim et al., 2018	29463073	nifedipine	10	Sprague-Dawley rat embryonic brain tissue	embryonic day 14	DMEM/F12 media with B27 supplement	24 hours	MTT Assay
Zhang et al., 2016	27541019	simvastatin	2, 5, 10, 25, 50	Sprague Dawley rat fetus	embryonic day 14.5	DMEM/F-12 with B27, BFGF, EGF, glutamine, heparin, penicillin streptomycin,	48 hours	WST-1
